# Clinical features and risk factors of plastic bronchitis caused by refractory Mycoplasma pneumoniae pneumonia in children: a practical nomogram prediction model

**DOI:** 10.1007/s00431-022-04761-9

**Published:** 2023-01-12

**Authors:** Han Zhang, Jingjing Yang, Wenqi Zhao, Jing Zhou, Shuangyu He, Yunxiao Shang, Qi Cheng

**Affiliations:** 1 Department of Pediatrics, Shengjing Hospital of China Medical University, 36Th Sanhao Street, Heping District, Shenyang, Liaoning 110004 People’s Republic of China; 2 Department of Pediatrics, Affiliated Hospital of Changchun University of Traditional Chinese Medicine, Changchun, Jilin, 130021 China; 3 Department of Pneumology, Xinmin People’s Hospital, Shenyang, 110300 Liaoning China

**Keywords:** Refractory Mycoplasma pneumoniae pneumonia, Plastic bronchitis, Fiberoptic bronchoscopy, Risk factors, Nomogram model, Prediction model

## Abstract

Early assessment of refractory Mycoplasma pneumoniae pneumonia (RMPP) with plastic bronchitis (PB) allows timely removal of casts using fiberoptic bronchoscopic manipulation, which relieves airway obstruction and limit sequelae development. This study aimed to analyze clinical data for risk factors and develop a nomogram for early predictive evaluation of RMPP with PB. The clinical data of 1-14 year-old patients with RMPP were retrospectively analyzed. Patients were classified into a PB or non-PB group. The general characteristics, clinical symptoms, laboratory test results, imaging findings, and microscopic changes of the two groups were compared. A statistical analysis of the risk factors for developing PB was performed, and a nomogram model of risk factors was constructed. Of 120 patients with RMPP included, 68 and 52 were in the non-PB and PB groups, respectively. Using multivariate logistic regression analysis, fever before bronchoscopy, extrapulmonary complications, pleural effusion, cough duration, and lactate dehydrogenase (LDH) levels were identified as risk factors. A nomogram was constructed based on the results of the multivariate analysis. The area under the receiver operating characteristic curve value of the nomogram was 0.944 (95% confidence interval: 0.779-0.962). The Hosmer-Lemeshow test displayed good calibration of the nomogram (p = 0.376, R2 = 0.723).

*Conclusion*: The nomogram model constructed in this study based on five risk factors (persistent fever before bronchoscopy, extrapulmonary complications, pleural effusion, cough duration, and LDH levels) prior to bronchoscopy can be used for the early identification of RMPP-induced PB.

**Table:** 

**What is Known:** *• Refractory Mycoplasma pneumoniae pneumonia (RMPP) in children has been increasingly reported and recognized, which often leads to serious complications.* *• Plastic bronchitis (PB) is considered to be one of the causes of RMPP, and bronchoscopic treatment should be improved as soon as possible to remove plastic sputum thrombus in bronchus.*	
**What is New:** *• This study determined the risk factors for RMPP-induced PB.* *• The nomogram model constructed in this study prior to bronchoscopy can be used for the early identification of RMPP-induced PB, which facilitate the early bronchoscopic removal of casts, thereby promoting recovery and reducing cases with poor RMPP prognosis.*

**What is Known:**

*• Refractory Mycoplasma pneumoniae pneumonia (RMPP) in children has been increasingly reported and recognized, which often leads to serious complications.*

*• Plastic bronchitis (PB) is considered to be one of the causes of RMPP, and bronchoscopic treatment should be improved as soon as possible to remove plastic sputum thrombus in bronchus.*

**What is New:**

*• This study determined the risk factors for RMPP-induced PB.*

*• The nomogram model constructed in this study prior to bronchoscopy can be used for the early identification of RMPP-induced PB, which facilitate the early bronchoscopic removal of casts, thereby promoting recovery and reducing cases with poor RMPP prognosis.*

## Introduction

*Mycoplasma* *pneumoniae* (MP) is a common lower respiratory tract pathogen that causes MP pneumonia (MPP) in children. The incidence of MP infection in cases of community-acquired pediatric pneumonia can be as high as 28 − 41% and is higher in school-aged children [1, 2]. In recent years, refractory MPP (RMPP) in children has been increasingly reported and recognized. RMPP is characterized by a high fever that does not subside or progressive changes on imaging despite conventional use of macrolide antibiotics for over 7 days [3, 4]. RMPP often leads to serious complications such as pleural effusion, pulmonary necrosis, and pulmonary embolism, and the patient is prone to sequelae, such as bronchitis obliterans (BO) or bronchiectasis.

Systemic glucocorticoid treatment can effectively suppress the excessive inflammatory response in RMPP and alleviate fever and pulmonary progression [5, 6]. However, some children respond poorly to treatment with conventional doses of glucocorticoids and continue to present with high fever and progression on imaging, exhibiting large consolidation shadows and pleural effusions. Bronchoscopy in such cases reveals the presence of plastic bronchitis (PB). In PB, the decrease in productive coughing leads to airway obstruction by viscous substances that travel through the bronchi and form casts. With the increase in RMPP cases and the introduction of bronchoscopy in the management of severe pediatric pneumonia, an increasing number of cases of RMPP combined with PB have been identified [7, 8]. The development of PB increases the difficulty of RMPP treatment [8]; therefore, pediatric respiratory physicians need to remove the casts as early as possible [9].

Thus, in this study, we retrospectively analyzed children who met the diagnostic criteria for RMPP and had underwent bronchoscopic examination. The patients were screened for the presence or absence of concomitant PB, and the clinical symptoms, laboratory test results, imaging findings, and microscopic changes of patients with and without PB were compared. This study determined the risk factors for RMPP-induced PB and constructed a nomogram to facilitate the early identification of PB from the clinical features of RMPP to facilitate the early bronchoscopic removal of casts, thereby promoting recovery and reducing cases with poor RMPP prognosis.

## Methods

### Study participants

Children with MPP, hospitalized in the Department of Pediatric Respiratory Medicine, Shengjing Hospital, China Medical University, between January 2015 and December 2019, who met the following diagnostic criteria for RMPP, were included in the study: (1) acute respiratory symptoms, including fever, coughing, or wheezing, with or without auscultatory abnormalities, such as bubbling sounds or diminished breath sounds; (2) chest CT findings suggestive of inflammatory infiltrates or consolidation; (3) positive serological test for MP-immunoglobulin M (IgM) and positive MP ribonucleic acid (RNA) test in nasopharyngeal swab sample or bronchoalveolar lavage fluid (BALF) [10]; and (4) persistent fever with axillary temperature ≥ 38.5 °C despite the conventional use of macrolide antibiotics for 7 or more days and persistent progression of clinical symptoms and chest imaging signs [10]. The exclusion criteria were as follows: (1) occurrence of chronic lung disease, bronchiectasis, BO, tuberculosis, liver or kidney disease, cardiovascular disease, or primary or secondary immune deficiency or (2) incomplete clinical data. This study was approved by the Institutional Review Board of Shengjing Hospital, China Medical University (approval number: 2016PS251K). Written informed consent was obtained from at least one guardian of each child prior to their participation in the study.

### Indications for bronchoscopy

In conjunction with clinical presentation and chest imaging, bronchoscopy was performed when MP infection with lobar consolidation or pulmonary atelectasis did not improve or progressed with treatment or when the diagnostic criteria for refractory pneumonia were met [11].

### Groups

Patients were classified into PB and non-PB groups based on their bronchoscopy results. The PB group exhibited partial obstruction of the bronchial lumen with sputum, which was removed using lavage, suction, and biopsy forceps, immersed in saline, and expanded into a cast with a “branching” texture. The non-PB group did not exhibit obstruction by sputum or BO; non-PB patients only presented with mucosal congestion, edema, rales, and flocculent sputum.

### Data collection

Patient data were collected when disease was in its acute phase. The following general clinical characteristics were collected: gender, age, duration of fever, temperature before and after bronchoscopy, cough status, presence of mixed infection, extrapulmonary organ involvement (including liver dysfunction, renal dysfunction, abnormal myocardial enzyme profile, and presence of abnormal neurological symptoms), whether macrolide antibiotics were used within 5 days of the course of the disease, whether hormones were used within 2 weeks of the course of the disease, and the course of hormone treatment. The following changes were determined using lung imaging: whether the area of pulmonary infiltration exceeded 2/3 of the lobe (Lung 23), pleural effusion, and site of consolidation pneumonia. The following laboratory analyses data were obtained: the level of white blood cells (WBCs), neutrophils, lymphocytes, C-reactive proteins (CRPs), lactate dehydrogenase (LDH), procalcitonin (PCT), D-dimer (DD), alanine aminotransferase (ALT), and albumin (ALB); presence of immune indicators (natural killer [NK] cell percentage, absolute NK count, T-helper cells/T-suppressor cells [CD4^+^/CD8^+^]) in peripheral blood; and CD4^+^/CD8^+^ ratio in bronchoalveolar lavage fluid (BALF), and cell fraction. Furthermore, data from blood MP-immunoglobulin M (IgM) testing, blood culture, nasopharyngeal swab, and bronchoalveolar alveolar lavage fluid tests for respiratory syncytial virus, influenza virus, adenovirus, parainfluenza virus, and MP-DNA and MP-RNA were obtained. Data from bronchoscopic findings, bronchial lumen sputum casts, occlusions, mucosal congestion and edema, and necrosis were also obtained. Children in the PB and non-PB groups were followed up by telephone to collect information on outcomes, including recurrent pneumonia, chronic cough, and recurrent wheezing within 1 year after diagnosis and followed up for imaging and bronchoscopic changes.

### Statistical analysis

SPSS software (V23.0, IBM, New York, USA) and R software (V.4.0.4, R Foundation for Statistical Computing, Vienna, Austria) were adopted for statistical analyses. The χ2 or Fisher’s exact test was used for categorical variables.

The skewed distribution data were expressed as the median (interquartile), and the Mann–Whitney *U* rank-sum test was used to compare the two groups. Variables with *p* < 0.1 were included in multifactor stepwise logistic regression to analyze the influencing factors of PB in patients with RMPP. The Forward: LR method was used to filter the variables. A nomogram was constructed based on the results of the previous multivariate analysis. The area under the receiver operating characteristic curve (AUC) and the Hosmer–Lemeshow goodness-of-fit test were used to evaluate the performance of the predictive model. A two-tailed *p* < 0.05 was considered statistically significant.

## Results

**Fig. 1:**
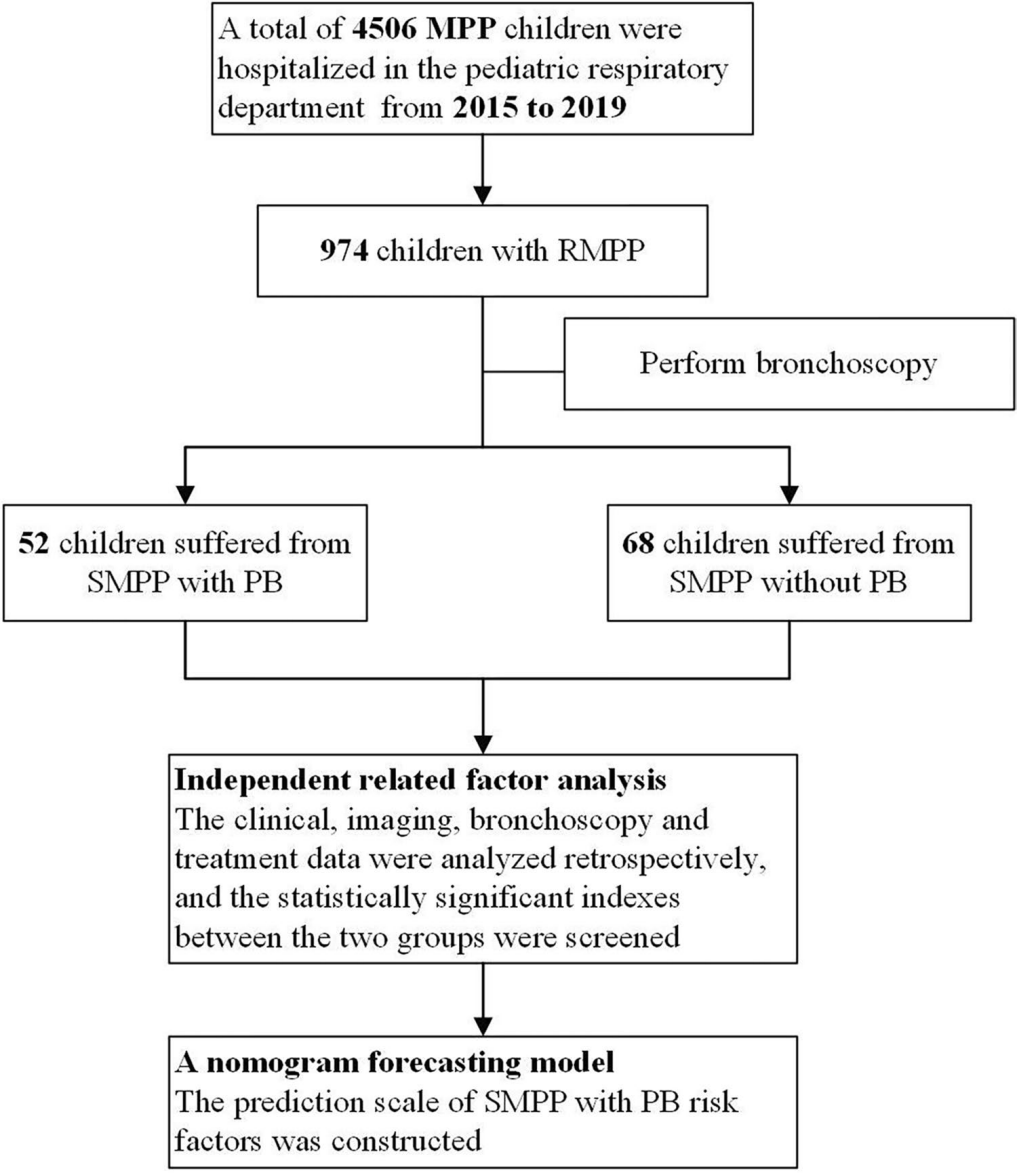
Flowchart of case screening and statistical analysis

Of the 4506 children diagnosed with MPP and hospitalized in the Department of Pediatric Respiratory Medicine of Shengjing Hospital, China Medical University, between January 2015 and December 2019, 974 were diagnosed with RMPP. Bronchoscopy was performed on the RMPP patients with large consolidation lesions or who failed drug treatment, of which 52 cases were in the PB group and 68 were in the non-PB group. Figure 1 shows the flowchart for the study.

### Analysis of general characteristics of cases of RMPP with PB

**Fig. 2:**
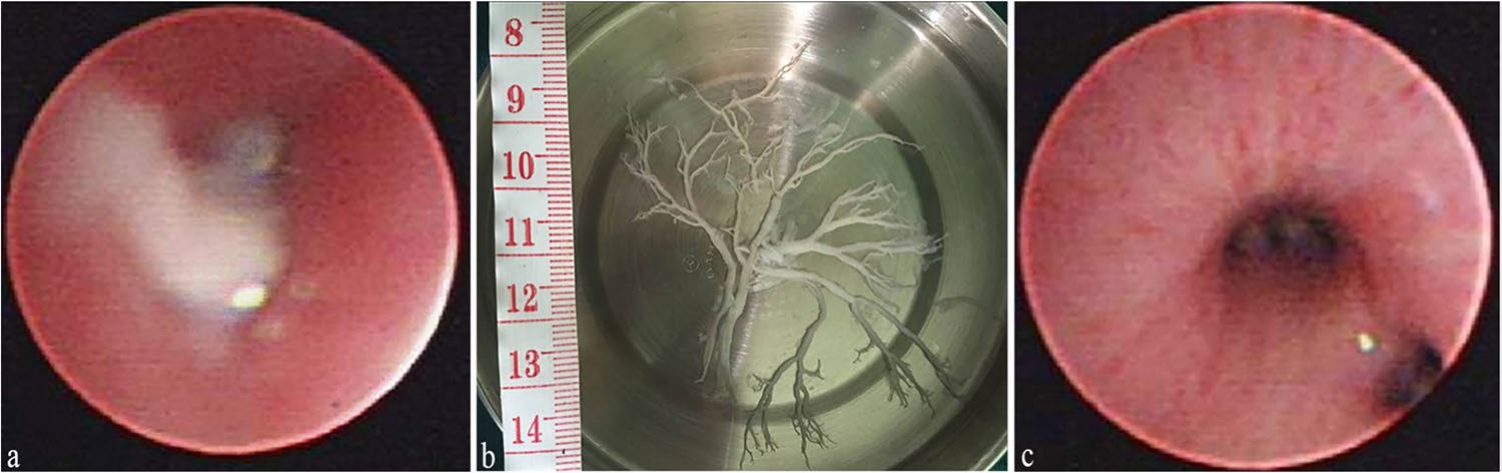
**a** Imaging changes before using fiberoptic bronchoscopy. **b** Bronchial cast removed under fiberoptic bronchoscopy. **c** Imaging changes after using fiberoptic bronchoscopy

Among the 52 patients with RMPP with PB, the time to detect PB was 10.44 ± 2.94 days. Forty-five children were febrile before bronchoscopy even after macrolide antibiotics and conventional hormone therapy. After bronchoscopy was performed to remove the casts, the temperature of most of the children returned to normal; only five remained febrile. Bronchoscopy revealed an open, white sputum occlusion (Fig. 2a), which was repeatedly flushed with acetylcysteine solution and normal saline (1:1 dilution). In patients for whom aspiration was difficult, biopsy forceps or foreign body retrieval baskets were used. The bronchial tree-shaped aspirated sputum plug was immersed in saline and unfolded into a bronchial tree-like cast 2 − 6 cm long (Fig. 2b). The bronchial lumen was clear after cleaning (Fig. 2c). There were 36 cases of repeat bronchoscopy; the efficacy of bronchoscopic treatment and repeat bronchoscopy are shown in Table 1. Of the 36 cases, 15 had sputum plug obstruction, 5 had mucosal necrosis, 20 had severe mucosal edema, 1 had BO, 5 had retained PB, and 17 cases had flocculent sputum (Table 1). Therefore, most PB cases required repeat bronchoscopy for complete airway clearance.

**Table 1:** Descriptive analysis of plastic bronchitis group

		Plastic bronchitis group
Timing of first bronchoscopy of disease course (days)		10.44 (± 2.94)
Presence of fever (before first bronchoscopy) (no/yes)		7/45
Presence of fever (after first bronchoscopy) (no/yes)		47/5
Timing of second bronchoscopy of disease course (*n* = 36)(days)		16.78 (± 6.03)
Results of second bronchoscopy	Sputum-thrombus (no/yes)	21/15
	Mucosal necrosis (no/yes)	31/5
	Edema (no/yes)	16/20
	Bronchial obliteration (no/yes)	35/1
	Plastic bronchitis (no/yes)	31/5
	Flocculus sputum (no/yes)	19/17

### Analysis of general and clinical characteristics of PB and non-PB groups

No difference in age or gender was observed between the PB and non-PB groups, and all children presented with fever and cough. The duration of cough was shorter in the PB group than in the non-PB group, and the duration of fever differed significantly between the two groups (*p* < 0.05). Although the duration of fever was shorter in the PB group than in the non-PB group, the number of fever cases before bronchoscopy was significantly higher in the PB group than in the non-PB group. Thus, the number of children with persistent fever after macrolide antibiotic and conventional hormone therapy before bronchoscopy was higher in the PB group than in the non-PB group (*p* < 0.05), suggesting a more severe systemic inflammatory response in PB.

There was a significant difference in the timing of the first bronchoscopy between the PB and non-PB groups (*p* < 0.05). The timing of the first microscopy was significantly earlier in the PB group (10.44 days) than in the non-PB group (14.39 days). No difference was observed between the timing (within 5 days of disease course) and total duration of macrolide antibiotic treatment and the timing (within 2 weeks of disease course) and total duration of hormone treatment. This result suggests that at approximately day 10 of disease course, if patients with RMPP with PB are febrile despite conventional macrolide antibiotic and hormone therapy, they should be promptly treated with bronchoscopy to remove sputum casts. These indicators may be risk factors for MP with PB (Table 2).

**Table 2:** Comparison of clinical characteristics and treatments

Factors	All (*n* = 120)	Non-PB group (*n* = 68)	PB group (*n* = 52)	Z/χ2	P
Gender (female/male)	63/57	38/30	25/27	0.72	0.396
Age (years)	5.9 (5.40, 6.40)	6.06 (5.40,6.73)	5.69 (4.89, 6.48)	0.534	0.466
Fever (days)	9.55 (8.84, 10.25)	10.16 (9.15, 11.16)	8.75 (7.78, 9.71)	3.922	0.05
Fever or not (before first bronchoscopy) (no/yes)	59/61	52/16	7/45	46.808	< 0.001
Cough	8.8 (8.04, 9.57)	10.25 (9.14, 11.35)	6.92 (6.13, 7.71)	21.281	< 0.001
Mix Infection (No/Yes)	67/53	42/26	25/27	2.239	0.135
Extrapulmonary complications (no/yes)	74/46	54/14	20/32	20.903	< 0.001
Timing of macrolides < 5 days of disease course (no/yes)	22/98	15/53	7/45	1.455	0.228
Total course of macrolides (days)	10.96 (9.95, 11.97)	10.89 (10.16, 11.63)	11.05 (8.88, 13.22)	0.024	0.877
Glucocorticoids (no/yes)	29/91	17/51	12/40	0.059	0.807
Total course of glucocorticoids (days)	5.72 (4.80, 6.64)	5.76 (4.52, 7.00)	5.67 (4.24, 7.09)	0.009	0.923
Treatment timing of glucocorticoids < 2 weeks (no/yes)	28/92	16/52	12/40	0.003	0.954
Timing of first bronchoscopy of disease course (days)	12.68 (11.91, 13.44)	14.39 (13.36, 15.43)	10.44 (9.62, 11.26)	32.581	< 0.001

There were significant differences in the level of laboratory indicators PCT, LDH, and ALT in the blood and CD4^+^ and CD8^+^ cells in the BALF between the PB and non-PB groups (*p* < 0.05). PCT, LDH, ALT, and BALF CD4^+^ levels were significantly higher in the PB group than in the non-PB group, and BALF CD8^+^ was significantly lower in the PB group than in the non-PB group. The incidence of pleural effusion was higher in the PB group than in the non-PB group, and the incidence of lesions involving over 2/3 of the lungs was lower in the PB group than in the non-PB group. The results indicate that the area of pneumonia was more limited and the local response was greater in the PB group than in the non-PB group (Table 3).

**Table 3:** Comparison of lab test and radiological features

Factors	All (*n* = 120)	non-PB group (*n* = 68)	PB group (*n* = 52)	Z/χ2	P
Blood test					
WBC ( *10^9/L)	9.88 (9.16, 10.61)	9.66 (8.72, 10.60)	10.17 (9.01, 11.34)	0.477	0.491
N%	63.22 (60.69, 65.75)	61.68 (58.93, 64.44)	65.23 (60.59, 69.88)	1.912	0. 169
L%	24.42 (22.15, 26.68)	25.15 (22.81, 27.48)	23.47 (19.13, 27.80)	0.526	0.47
CRP (mg/L)	30.74 (23.50, 37.97)	30.93 (22.44, 39.41)	30.49 (17.66, 43.32)	0.003	0.953
LDH (U/L)	462.53 (422.65, 502.41)	413.3 (372.52, 454.09)	526.9 (453.87, 599.92)	8.291	0.005
PCT (ng/mL)	0.31 (0.22,0.40)	0.18 (0.13,0.22)	0.49 (0.29,0.68)	12.004	0.001
D-dimer (ug/L)	1295.69 (1033.56, 1557.81)	1153.94 (794.15, 1513.73)	1481.05 (1093.04, 1869.07)	1.506	0.222
ALT (U/L)	39.61 (30.08, 49.14)	29.22 (23.27, 35.17)	53.21 (32.83, 73.59)	6.377	0.013
ALB (g/L)	36.15 (35.29, 37.00)	36.62 (35.40, 37.85)	35.52 (34.33, 36.71)	1.609	0.207
Lymphocyte subsets					
NK%	10.94 (9.57, 12.31)	11.25 (9.48, 13.02)	10.54 (8.31, 12.77)	0.254	0.615
Count of NK cells (/ul)	229.21 (191.49, 266.93)	222.94 (176.52, 269.35)	237.42 (173.21, 301.63)	0.141	0.708
CD4 ^+^ /CD8 ^+^	1.48 (1.35, 1.61)	1.39 (1.23, 1.56)	1.6 (1.39, 1.81)	2.455	0.12
BALF test					
Lymphocyte subsets					
NK %	12.14 (10.39, 13.89)	11.27 (9.14, 13.40)	13.27 (10.29, 16.25)	1.261	0.264
CD4 ^+^ %	23.42 (20.95, 25.90)	20.87 (17.51, 24.23)	26.76 (23.19, 30.33)	5.672	0.019
CD8 ^+^ %	51.99 (48.82, 55.15)	56.82 (52.73, 60.91)	45.66 (41.11, 50.22)	13.202	< 0.001
Percentage of lobulated nuclear cells	39.56 (35.24, 43.88)	41.85 (36.08, 47.61)	36.57 (29.91, 43.24)	1.439	0.233
Radiologic features					
Lung infiltration area more than2/3of lung (no/yes)	63/57	30/38	33/19	4.421	0.035
Effusion (no/yes)	80/40	54/14	26/26	11.471	< 0.001

*WBC* white blood cell, *N* neutrophil, *L* lymphocyte, *CRP* C-reactive protein, *LDH* lactate dehydrogenase, *PCT* procalcitonin, *ALB* albumin, *ALT* alanine aminotransferase, *NK* natural killer cells, *BALF* bronchoalveolar lavage fluid

### Multivariate regression analysis of PB in RMPP

A multifactorial analysis with the possible influencing factors as the independent variables and the development of PB as the dependent variable was performed to determine the independent factors influencing the development of PB in the context of RMPP. The results showed that the cough duration in days, presence of fever before bronchoscopy, extrapulmonary complications, pleural effusion, and LDH were independent factors influencing the development of PB with RMPP (Table 4).

**Table 4:** Multivariate analysis

Factors	B	S.E.	Wald Z	*p*-value	Exp (B)	95% CI	
						floor	upper
Cough	−0.379	0.106	-3.57	0.0004	0.684	0.556	0.843
Presence of fever (before first bronchoscopy)	4.385	0.947	4.63	< 0.0001	80.213	2.547	512.799
Extrapulmonary complications (No/Yes)	3.14	0.877	3.58	0.0003	23.1	4.138	128.972
Effusion (no/yes)	1.818	0.753	2.42	0.016	6.16	1.409	26.925
LDH (U/L)	−0.004	0.002	-2.09	0.037	0.996	0.992	1
constant	0.170	0.964	0.18	0.86	1.185		

### PB occurrence nomogram and nomogram performance evaluation

**Fig. 3:**
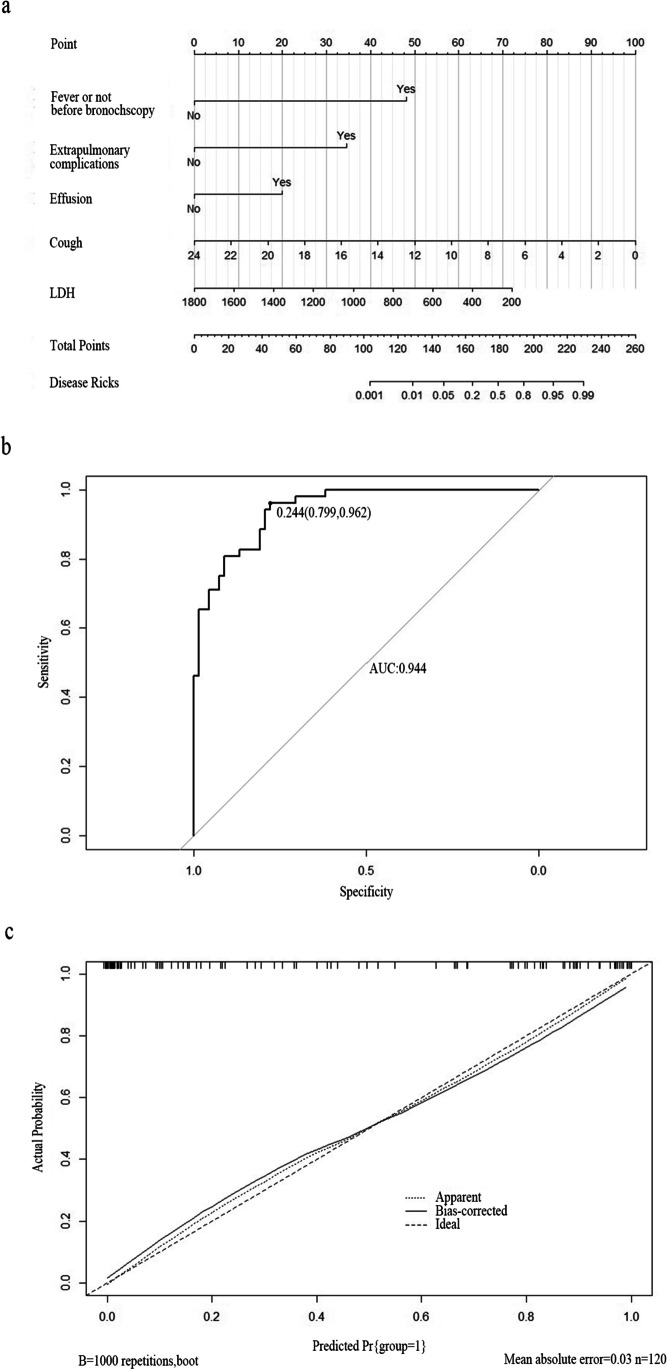
**a** Nomogram of regression equations for calculating risk score and predicting risk of PB in RMPP patients. **b** Receiver operating characteristic (ROC) curve analysis of main cohort. **c** Nomogram calibration curve. Horizontal axis indicates risk of PB occurrence predicted by nomogram. Vertical axis represents actual observed risk of PB occurrence (presence of fever before first bronchoscopy, ExLung, effusion, cough, LDH)

A nomogram of the risk of PB was plotted using the five risk factors obtained from the results of the logistic regression analysis (Fig. 3a). The AUC of the nomogram was 0.944. The nomogram was generated by assigning a weighted score to each independent influencing factor. The highest score is 100 points, and the range of PB incidence is 0.01 to 0.99. A higher score calculated from the sum of the distribution points of each high-risk factor in the nomogram corresponds to a higher risk of occurrence. The Hosmer–Lemeshow test was adopted for the model test, and the result was *p* = 0.376, R^2^ = 0.723, indicating that the information in the current data had been fully extracted. The AUC value showed that the predictive power of the predictive model in the main cohort was 0.944 (95% CI 0.779–0.962) (Fig. 3b). The calibration chart showed that the nomogram had a sufficient fit for predicting PB incidence in RMPP patients (Fig. 3c).

### Comparison of prognosis and sequelae between PB and non-PB groups

The PB and non-PB groups were followed up by telephone, and post-discharge follow-up data were collected. After excluding the cases that were not reexamined or lost to follow-up, 98 cases remained, of which 30 and 68 cases were in the PB and non-PB groups, respectively (Table 5). All 68 cases in the non-PB group exhibited recovery on imaging and did not develop BO; of the 30 cases in the PB group, 7 did not exhibit recovery on imaging and 6 cases progressed to BO. Therefore, incomplete recovery on imaging and the development of BO are sequelae of PB. The present and previous studies suggest that PB is a high-risk factor for the future development of BO [12]. Additionally, the incidence of subsequent recurrent pneumonia was higher in the PB group than in the non-PB group, suggesting more local mucosal injury and immune dysfunction in the bronchi of the PB group, which is also associated with the imbalanced CD4^+^/CD8^+^ ratio described earlier. Therefore, bronchial casts can form repeatedly and require multiple bronchoscopies in some patients. The results suggest that early identification of factors influencing the development of PB in children with RMPP and early refinement of bronchoscopy may be beneficial in relieving clinical symptoms.

**Table 5:** Comparison of sequelae

	Factors	All (*n* = 98)	Non-PB group (*n* = 68)	PB group (*n* = 30)	χ2	P
Sequela	Chronic cough (no/yes)	94/4	65/3	29/1	0.062	0.804
	Recurrent pneumonia (no/yes)	92/6	67/1	25/5	8.363	0.004
	Recurrent wheezing (no/yes)	96/2	67/1	29/1	0.361	0.548
	Asymptomatic (no/yes)	11/87	5/63	6/24	3.341	0.068
Prognosis	Recovery (no/yes)	7/91	0/68	7/23	17.087	< 0.0001
	BO (no/yes)	92/6	68/0	24/6	14.487	< 0.0001
	Atelectasis (no/yes)	97/1	68/0	29/1	2.29	0.13

## Discussion

PB has been previously reported as a rare respiratory disease in children in which a jelly-like or stiff bronchial fluid travels rapidly in a tube-shaped pattern along the bronchial tree through the airways, obstructing them and causing clinical symptoms such as shortness of breath and dyspnea that can be life-threatening in severe cases [13, 14]. However, its etiology is still poorly understood. Previous studies have reported possible associations with postoperative congenital heart disease [15–17], asthma [18], bronchial thermoplasty [19], congestive heart failure [20], lung transplantation [21], and acute respiratory viral infections [22]. PB is also found in AIDS patients with pulmonary Kaposi sarcoma [23] and in rare cases, after lung transplantation [21]. Congenital heart disease leads to decreased cardiac function and increased pulmonary venous pressure, which increases mucus secretion and leakage of endobronchial lymph in the airways, resulting in cast formation [9, 24, 25]. Viral infections may cause a hypersecretory state in bronchial endothelial cells, leading to bronchial cast formation [22, 26, 27]. The literature reports that the incidence and mortality rates of PB are 6.8/100,000 and 7%, respectively [20]. Recently, the number of cases of PB caused by MP infection has steadily increased [8], which may be related to the epidemiological characteristics of MP in Asia [28]. The clinical manifestations of PB caused by MP infection are not specific and are often difficult to distinguish from RMPP without PB, as both present with fever, cough, and large consolidation shadows in the chest that make it difficult to distinguish them based on symptoms, signs, and imaging findings. The diagnosis and treatment of PB require fiberoptic bronchoscopy as early as possible [9]; therefore, the timely detection of PB in the context of RMPP using clinical data is particularly important. The mechanism of PB development after MP infection remains unclear and may be closely associated with MP drug resistance and excessive immune response [5]. PB caused by MP is associated with abnormally elevated levels of the cytokines IL-1β, IL-8, IL-2, and IL-10 [8], and ribosomal RNA-depleted RNA sequencing in RMPP [29]. Currently, there is no consensus on the etiology and pathology of PB, and further studies are required.

Comparison and analyses of the PB and non-PB group data indicated that the duration of fever was less in the PB group than in the non-PB group; however, more cases of fever were observed before bronchoscopic treatment in the PB group than in the non-PB group. Additionally, the timing of bronchoscopy treatment was earlier in the PB group than in the non-PB group, and the fever in the PB group was significantly relieved after bronchoscopy. Most cases of cast removal are associated with immediate relief of respiratory obstruction and inflammatory response [30, 31]. Cough duration was less in the PB group than in the non-PB group, and our clinical observations indicated that children with PB had a less pronounced cough. It is unclear whether this was owing to a weakened cough reflex that was not conducive to sputum elimination or to a severe inflammatory response that promoted sputum coagulation and PB, leading to a weakened cough. The frequencies of extrapulmonary complications, pleural effusion, LDH, and the immune indicator CD4^ +^ were higher in the PB group than in the non-PB group, suggesting that the local immune response and systemic immune-inflammatory response were stronger in the MP-induced PB cases than in the non-PB cases. The incidence of lesions involving more than 2/3 of the lungs was lower in the PB group than in the non-PB group, suggesting that pneumonia in the PB group was smaller and more localized. Additionally, plastic casts were mostly confined to one lung segment, with uniform and consistent solid lung lesions rather than exudate involvement along the bronchial routes of multiple lobe segments. Pleural effusion resulted from severe local inflammation, luminal occlusion, and increased local hydrostatic pressure.

Additionally, the present study found that most cases of PB due to MP were relatively mild with no life-threatening manifestations, in contrast to previous reports of PB caused by viral infections [22, 26, 31]. However, untimely removal of casts may lead to an increased incidence of poor prognosis in RMPP. We found a correlation between PB and BO [12], and some children had sputum casts even at the second or third bronchoscopy.

Unlike previous single-factor analyses of risk factors for PB [8], this study employed a multifactor analysis of the risk factors for developing PB in RMPP. Additionally, a nomogram was constructed, in which each risk factor was assigned a score, thus allowing them to be quantified. The scores corresponding to the risk factors can be added, and the total can be used to predict the risk of PB in children with RMPP. In this study, blood LDH level, which is currently receiving increasing attention in diagnosing and predicting RMPP [4, 32, 33], was significantly higher in the PB group than in the non-PB group. It is significantly elevated in children with RMPP who develop PB and is also a risk factor for developing PB [8], consistent with the results of the present study. LDH level was a factor in the nomogram developed in this study. Each high-risk factor in the nomogram is assigned a score. Whether this indicator gradually increases or decreases in the nomogram is governed by many factors, and it is necessary to differentiate it from the correlation of the quantitative tendency of the development of PB. The scores assigned to each indicator are added to determine the total score, which can be used to predict the probability of the development of PB in RMPP.

Regardless of the etiology, the most important treatment for PB is the removal of casts via fiberoptic bronchoscopy [9, 34]. Treatments with 3% hypertonic saline and bronchodilators have also been reported [35]. Furthermore, reports describe physical therapy with high-frequency chest wall oscillation, nebulized urokinase, local irrigation with tissue plasminogen activator (t-PA) [21, 36], and recombinant human deoxyribonuclease [35]. A combination of local t-PA and cryotherapy has also been used [37]. The use of nebulized heparin inhalation [9, 34], acetylcysteine, and diuretics [38] has been reported. Because different primary diseases have varying mechanisms of cast formation and differences in disease severity and incidence, PB is mostly mentioned in the literature in case reports. PB is also treated with a combination of intravenous drugs; therefore, it is difficult to determine which drug approach is the most effective. However, fiberoptic bronchoscopy is now accepted as the most effective technique for removing casts from the airways.

In the present study, sputum casts were cleared using fiberoptic bronchoscopy combined with repeated saline flushing or diluted acetylcysteine solution (1:1 dilution) for lavage aspiration or using biopsy forceps or foreign body retrieval baskets. In all cases, the pathogen detected was MP, and the patients were treated with intravenous macrolide antibiotics and administered intravenous methylprednisolone [6, 39]. These treatments have been correlated with MP resistance and an excessive inflammatory response [5, 6, 28, 39]. The primary sequela of MP-induced plastic bronchitis is BO [12]. Early diagnosis and treatment and repeated bronchoscopic flushing to completely clear the airway in some cases may result in an improved prognosis. This study was a retrospective data analysis. A prospective study is proposed to investigate the patterns of this disease.

## Conclusions

In summary, our research has developed a nomogram with five factors, including fever or not before bronchoscopy, extrapulmonary complications, effusion, cough, and LDH with the purpose to predict the risk of PB in children due to RMPP. The nomogram has performed well and may help the clinical identification and decision-making of PB patients caused by RMPP.

## Data Availability

The datasets generated and/or analyzed during the current study are available from the corresponding author on reasonable request.

## Abbreviations

MP: *Mycoplasma pneumoniae*
MPP: Mycoplasma pneumoniae pneumonia
RMPP: Refractory Mycoplasma pneumoniae pneumonia
PB: Plastic bronchitis
BO: Bronchitis obliterans
IgM: Immunoglobulin M
DD: D-dimer
NK: Natural killer
t-PA: Plasminogen activator
WBC: White blood cell
CRP: C-reactive protein
LDH: Lactate dehydrogenase
PCT: Procalcitonin
ALB: Albumin
ALT: Alanine aminotransferase
BALF: Bronchoalveolar lavage fluid
ExLung: Extrapulmonary complication
Lung23: Consolidation of more than 2/3 of the lungs
AUC: Area under the receiver operating characteristic curve
